# Assessment of Satisfaction With the Electronic Health Record Among Physicians in Physician-Owned vs Non–Physician-Owned Practices

**DOI:** 10.1001/jamanetworkopen.2022.8301

**Published:** 2022-04-21

**Authors:** Lisa S. Rotenstein, Nate Apathy, Bruce Landon, David W. Bates

**Affiliations:** 1 Department of Medicine, Brigham and Women’s Hospital, Boston, Massachusetts; 2 Department of Medicine, Harvard Medical School, Boston, Massachusetts; 3 Leonard Davis Institute of Health Economics, Wharton School, Philadelphia, Pennsylvania; 4 Department of Medicine, Perelman School of Medicine, Philadelphia, Pennsylvania; 5 Regenstrief Institute, Indianapolis, Indiana; 6 Department of Healthcare Policy, Harvard Medical School, Boston, Massachusetts; 7 Department of Medicine, Beth Israel Deaconess Medical Center, Boston, Massachusetts; 8 Department of Health Policy and Management, Harvard School of Public Health, Boston, Massachusetts

## Abstract

**Importance:**

Despite known benefits, electronic health records (EHRs) have had drawbacks for daily practice and the physician experience. There is evidence that physicians practicing in solo or physician-owned practices are more likely to be satisfied with the EHR and experience lower burnout than those practicing in other ownership arrangements; however, it is unclear how practice ownership patterns interact with physicians’ experiences with the EHR and documentation in the EHR now that use of these systems is widespread.

**Objective:**

To examine the association between practice ownership and physician perceptions of the EHR.

**Design, Setting, and Participants:**

This cross-sectional study included non–federally employed physicians who provided office-based patient care in 2019 and completed the 2019 National Electronic Health Records Survey. The 2019 survey sample consisted of 1524 eligible responses (41.0% unweighted response rate representing 301 603 physicians); of those, 1368 physicians who reported having an EHR and answered questions regarding location ownership were included in the analysis. Data for the 2019 National Electronic Health Records Survey were collected by RTI International from June 14 to December 11, 2019; the current cross-sectional analysis was conducted from October 1 to November 30, 2021.

**Main Outcomes and Measures:**

Satisfaction with the EHR, perceptions of time spent on clinical documentation, and presence of staff support for documentation.

**Results:**

Among 1368 respondents (weighted, 270 813 respondents) included in the analysis, 960 respondents (weighted: 185,385 respondents [68.5%]) were male, and 951 respondents (weighted: 200,622 respondents [74.1%]) were over 50 years of age; 766 respondents (weighted, 161 226 respondents [59.5%]) were working in a practice owned by a physician or physician group, and 700 respondents (weighted, 131 284 respondents [48.5%]) were primary care physicians. A total of 602 respondents (weighted, 109 587 physicians [40.5%]) were working in a non–physician-owned practice. Overall, 529 respondents (weighted, 108 093 respondents [68.1%]) working in physician-owned practices reported being satisfied with their EHR vs 320 respondents (weighted, 63 988 respondents [58.5%]) working in non–physician-owned practices (*P* = .03). Among those working in physician-owned practices, perceptions that time spent on documentation was appropriate (328 physicians [weighted, 71 827 physicians (44.8%)] vs 191 physicians [weighted, 35 447 physicians (32.4%)]; *P* = .005) and that staff support for documentation was available (289 physicians [weighted, 57 702 physicians (36.0%)] vs 146 physicians [weighted, 29 267 physicians (26.7%)]; *P* = .02) were significantly higher compared with those working in non–physician-owned practices. Physicians’ perceptions of the appropriateness of time spent and the availability of staff support only partially explained the association between practice ownership type and EHR satisfaction.

**Conclusions and Relevance:**

The results of this nationally representative cross-sectional study suggest that physicians working in physician-owned practices are more likely to be satisfied with the EHR, to have positive perceptions of time spent on documentation, and to have staff support for documentation compared with their counterparts working in non–physician-owned practices. The workflow and cultural forces underlying these differences are important to understand in the setting of known differences in burnout by practice ownership type and ongoing physician group consolidation and acquisition by health care systems.

## Key Points

**Question:**

Is practice ownership associated with physician perceptions of the electronic health record (EHR)?

**Findings:**

In this cross-sectional study of 1368 physicians who responded to the 2019 National Electronic Health Records Survey, EHR satisfaction, perception that time spent on documentation was appropriate, and the presence of staff support for documentation were significantly higher among those working in physician-owned practices compared with non–physician-owned practices. Differences in physician perceptions of the appropriateness of documentation time and the presence of staff support for documentation only partially explained the differences in EHR satisfaction by practice ownership.

**Meaning:**

The study’s findings suggest that multiple features associated with physician ownership of practices are likely to play a role in greater EHR satisfaction.

## Teaser

This cross-sectional study uses data from the 2019 National Electronic Health Records Survey to examine the association between physician perceptions of the electronic health record and practice ownership type.

## Introduction

The introduction and increased use of electronic health records (EHRs), bolstered by the Health Information Technology for Economic and Clinical Health (HITECH) Act,^1^ has had many benefits for clinical practice and quality. Although findings are mixed, use of EHRs has been associated with improved chronic disease management,^2^ decreased rates of adverse events,^3,4,5^ facilitation of team-based care,^6^ and improved quality of care.^7^ A primary goal of the HITECH Act was to lay the digital foundation to improve health care quality, but it is not clear whether quality has changed broadly.

The advent of EHRs has also had drawbacks for daily practice and the physician experience. Previous research^8,9^ has found an association between increased time spent using the EHR and burnout. In a 2016 study^10^ of EHR optimization, 70% of a national sample of physicians attributed an increase in their administrative burdens to the EHR. Time spent using the EHR varies substantially across specialties^11,12^ and in US vs non-US settings.^13^ In some settings, clinician computer use during a visit has been associated with lower patient satisfaction and communication challenges.^14^ Notably, when using a standardized product usability scale, physicians have rated the usability of the EHR in the not acceptable range and in the bottom 9% of scores across previous studies^15,16^; specifically, physicians have rated EHR usability lower than that of Microsoft Excel software, which has the lowest rating among commonly used electronic consumer products.^17^ Usability of the EHR, which incorporates the concept of product satisfaction, has been independently associated with greater odds of burnout among physicians,^15^ and experience with EHRs has been associated with physicians’ professional satisfaction.^18^

Previous research^18,19,20^ has also found an association between the practice’s structural characteristics and physician well-being. For instance, physicians practicing in solo or physician-owned practices have lower rates of burnout^19^ and are more satisfied,^18^ and solo and physician-owned practices may be more likely to adopt leadership behaviors that support physician satisfaction and well-being.^20^ However, fewer physicians have been practicing in physician-owned practices over time. For example, a recent national report^21^ found a 12% increase in physicians employed by hospital systems or other corporate entities between 2019 and 2021.

It is possible that physician-owned practices may also be better equipped to involve physicians in the design and implementation of EHRs, control trade-offs with the EHR (eg, between take-home pay and investment in resources to aid documentation), and directly reap the benefits of EHRs. However, we know little about which organizational or setting-specific factors are associated with higher satisfaction with health care information technologies. In combination with knowledge about organizational design factors associated with lower burnout,^19^ understanding the association between practice ownership and EHR experience may help to shape policy and systems interventions that enhance quality of care and the physician experience across organizational settings. Using data from the National Electronic Health Records Survey (NEHRS), we sought to examine the association between practice ownership and physician perceptions of the EHR.

## Methods

We performed a cross-sectional study of the association between practice ownership characteristics and physician perceptions of their EHR experience using data from the 2019 NEHRS. The NEHRS is an annual survey that queries a sample of non–federally employed physicians who provide office-based patient care in the US and are principally engaged in patient care activities; the names of physicians invited to participate in the NEHRS are derived from the master files of the American Medical Association and the American Osteopathic Association. Per the Common rule, this study was exempt from institutional review board review and informed consent because of its use of publicly available, deidentified data. The study followed the Strengthening the Reporting of Observational Studies in Epidemiology (STROBE) reporting guidelines for cross-sectional studies.

### Survey Administration and Sample

The NEHRS is administered annually by the National Center for Health Statistics. Data for the 2019 NEHRS were collected by RTI International from June 14 to December 11, 2019.^22^ The survey was administered via a web-based instrument submitted electronically, a paper-based instrument submitted via the mail, or a telephone survey via a computer-assisted telephone interview. Among eligible physicians, 1524 (weighted, 301 603) completed the 2019 survey, representing an unweighted response rate of 41.0%.^22^ The current cross-sectional analysis was conducted between October 1 to November 30, 2021.

### Measures

#### Practice Factors

The NEHRS queries physicians about their specialty, the type of practice setting in which they provide care for ambulatory patients, the ownership of the practice in which they are employed, the number of physicians in the practice setting, and the practice’s participation or nonparticipation in a value-based payment arrangement (accountable care organization [ACO] or advanced alternative payment model). Specialties are categorized as medical, surgical, or primary care. We categorized practice ownership as physician-owned (comprising practices owned by independent physicians or physician-owned groups) or non–physician-owned (comprising practices owned by an insurance company; a health maintenance organization, health care plan, or other health care corporation; a community health center; or a medical or academic health care center or other hospital).

The NEHRS then asks physicians whether their practice location uses an EHR system; those who answer affirmatively are asked to provide the name of their primary EHR system vendor. Given the focus of our study, the analysis was limited to physicians who reported that their practice location used an EHR system. Based on the relative prevalence of EHRs nationwide,^23^ we categorized the EHR vendors into Allscripts (Allscripts Healthcare Solutions, Inc), athenahealth (athenahealth, Inc), Cerner (Cerner Corporation), eClinicalWorks (eClinicalWorks, LLC), Epic (Epic Systems Corporation), NextGen (NextGen Healthcare, Inc), Practice Fusion (Practice Fusion, Inc), or other (consisting of Amazing Charts [Amazing Charts, LLC], e-MDs [CompuGroup Medical], GE/Centricity [GE Healthcare], Modernizing Medicine [ModMed], Sage/Vitera/Gateway [Sage Intergy; Vitera Healthcare Solutions, LLC; and Gateway Electronic Medical Management Systems], or a choice not provided on the survey).

#### EHR-Related Experiences and Support

The NEHRS asks respondents about their perceptions of documentation functions, the burden associated with their medical record system, and the staff support they receive for documentation. Respondents are specifically asked how satisfied they are with their EHR and how easy or difficult it is to document clinical care using their medical record system. They are also asked about the extent to which they agree that the amount of time spent documenting clinical care is appropriate, that the time spent on documentation does not reduce time spent with patients, and that the documentation required solely for billing but not clinical purposes increases the amount of time spent on documentation. Responses were categorized as *very satisfied* or *somewhat satisfied* vs *neither dissatisfied nor satisfied*, *somewhat dissatisfied*, or *very dissatisfied*. We similarly grouped those who reported documentation in their medical record system as *very easy* or *somewhat easy* and those who *strongly agreed* or *somewhat agreed* with statements regarding the appropriateness of time spent documenting clinical care, the impact of documentation for time spent with patients, and the impact of billing documentation for overall time spent documenting clinical care. Respondents were queried about how many hours they spent outside of normal office hours documenting clinical care in their medical record system (with choices including none, <1 hour, 1 to 2 hours, >2 to <4 hours, and >4 hours) and whether they had staff support (eg, a scribe) to assist with documenting clinical care.

### Statistical Analysis

We first analyzed the sample descriptively in terms of practice ownership, physician specialty, number of physicians in the practice, and EHR vendor used. We further described the distribution of specialty, number of physicians, EHR vendor used, and value-based payment arrangement status (ACO or advanced alternative payment model) by practice ownership.

We compared responses to questions regarding EHR satisfaction, ease of documentation, staff support for documentation, time spent using the EHR, and perceptions of documentation impact by practice ownership using χ^2^ tests. We also stratified bivariate analyses by specialty. We then used multivariable logistic regression models with clustering at the physician level (as specified by the National Center for Health Statistics) to examine the association between ownership type and EHR satisfaction, adjusted for specialty and participation or nonparticipation in an ACO (with practice size and EHR vendor not included as covariates in our main model given their collinearity with ownership). We subsequently explored the extent to which nonsatisfaction perceptions that differed significantly by ownership type explained the association between ownership and EHR satisfaction.

In sensitivity analyses, we examined the association between EHR vendor and EHR satisfaction. We then adjusted for EHR vendor in our base model and in models with additional covariates to assess the extent to which the association between practice ownership and EHR satisfaction was confounded by differences in EHR vendor across physician-owned vs non–physician-owned practices.

All analyses were conducted using SAS OnDemand for Academics (SAS Institute, Inc), with a 2-sided significance threshold of *P* = .05 using prespecified NEHRS subpopulation procedures. Sampling weights used in the NEHRS account for differences in selection probability by state and specialty group, and physician-level estimation weights specified by the NEHRS were used to produce national estimates.

## Results

### Sample

Our analysis included 1368 respondents (89.8% of physicians) who reported having an EHR and answered questions regarding location ownership, representing a weighted sample of 270 813 physicians; 960 respondents (weighted: 185,385 respondents [68.5%]) were male, and 951 respondents (weighted: 200,622 respondents [74.1%]) were over 50 years of age. (Race and ethnicity were not addressed in the data sample, and we could not provide any data on the race or ethnicity of physicians in the sample.) A total of 766 respondents (weighted, 161 226 respondents [59.5%]) reported working in a practice owned by a physician or physician group, and 700 respondents (weighted, 131 284 respondents [48.5%]) were primary care physicians (Table 1). Of 602 physicians (weighted, 109 587 physicians [40.5%]) working in a non–physician-owned practice, 183 physicians (weighted, 37 818 physicians [34.5%]) worked in a medical or academic health care center. Overall, 958 physician-owned and non–physician-owned practices (weighted, 196 585 practices [72.6%]) employed 10 or fewer physicians.

**Table 1.** Participant Characteristics

Characteristic	Unweighted, No.	Weighted, No. (%)
Total participants, No.	1368	270 813
Ownership of practice		
Physician-owned	766	161 226 (59.5)
Non–physician-owned	602	109 587 (40.5)
Specialty		
Primary care	700	131 284 (48.5)
Surgical	300	59 439 (21.9)
Medical	368	80 089 (29.6)
No. of physicians in practice		
1	262	54 333 (20.1)
2-3	248	54 609 (20.2)
4-10	448	87 643 (32.4)
11-50	244	41 055 (15.2)
>50	165	33 161 (12.2)
EHR vendor		
Allscripts	105	19 682 (7.3)
athenahealth	88	15 162 (5.6)
Cerner	103	17 272 (6.4)
eClinicalWorks	125	27 848 (10.4)
Epic	319	64 603 (24.1)
NextGen	71	14 933 (5.6)
Practice Fusion	37	12 054 (4.5)
Other	510	97 002 (36.1)
Participation in value-based payment model		
Accountable care organization	492	94 485 (34.9)
Advanced alternative payment model	125	21 428 (7.9)

Abbreviation: EHR, electronic health record.

Across all respondents, Epic was the most commonly used EHR vendor (319 physicians; weighted, 64 603 physicians [24.1%]) followed by eClinicalWorks (125 physicians; weighted, 27 848 physicians [10.4%]). Notably, 510 surveyed physicians (weighted, 97 002 physicians [36.1%]) reported using an EHR vendor that was not included among the survey choices (Table 1). In physician-owned practices, EHR choices were dispersed across vendors, with slightly more physicians using eClinicalWorks (96 physicians; weighted, 19 192 physicians [12.1%]) and Epic (68 physicians; weighted, 15 354 physicians [9.6%]). In contrast, 251 physicians (weighted, 49 249 physicians [45.0%]) working in non–physician owned practices reported using Epic (Table 2). Physician-owned practices were more likely to have 1 to 3 physicians (368 respondents; weighted, 83 991 respondents [52.1%]), whereas non–physician-owned practices were more likely to have 11 or more physicians (255 respondents; weighted, 43 455 respondents [39.7%]). Non–physician-owned practices were more likely to participate in an ACO (247 respondents; weighted, 45 938 respondents [41.9%]) (Table 2). Additionally, EHR choice varied by practice size; 93 physicians (weighted, 19 560 physicians [60.5%]) working in a practice with 50 or more physicians used Epic. In contrast, among practices with 1 to 3 physicians, no single EHR was used by more than 14% of the sample, with *other EHR* (ie, an EHR not listed) being the most common selection.

**Table 2.** Participant Characteristics by Practice Ownership

Characteristic	Physician-owned		Non–physician-owned		*P* value
	Unweighted, No.	Weighted, No. (%)	Unweighted, No.	Weighted, No. (%)	
Total participants, No.	766	161 226	602	109 587	NA
Specialty					
Primary care	357	66 402 (41.2)	343	64 882 (59.2)	<.001
Surgical	192	43 110 (26.7)	108	16 330 (14.9)	
Medical	217	51 714 (32.1)	151	28 376 (25.9)	
No. of physicians in practice					
1	206	45 419 (28.2)	56	8913 (8.1)	<.001
2-3	162	38 572 (23.9)	86	16 037 (14.6)	
4-10	244	46 473 (28.8)	204	41 170 (37.6)	
11-50	107	21 025 (13.0)	137	20 030 (18.3)	
>50	47	9736 (6.0)	118	23 425 (21.4)	
EHR vendor					
Allscripts	73	14 436 (9.1)	32	5247 (4.8)	<.001
athenahealth	53	11 149 (7.0)	35	4013 (3.7)	
Cerner	12	1340 (0.8)	91	15 932 (14.6)	
eClinicalWorks	96	19 192 (12.1)	29	8657 (7.9)	
Epic	68	15 354 (9.6)	251	49 249 (45.0)	
NextGen	53	10 073 (6.3)	18	4860 (4.4)	
Practice Fusion	32	8624 (5.4)	5	3429 (3.1)	
Other	371	79 032 (49.6)	139	17 969 (16.4)	
Participation in value-based payment model					
Accountable care organization	245	48 547 (30.1)	247	45 938 (41.9)	.006
Advanced alternative payment model	70	13 029 (8.1)	55	8399 (7.7)	.86

Abbreviations: EHR, electronic health record; NA, not applicable.

### EHR Experiences by Practice Ownership

In unadjusted analyses, EHR satisfaction, staff support for documentation, and perceptions of the appropriateness of time spent on documentation were significantly different by practice ownership (Table 3). A total of 529 respondents (weighted, 108 093 respondents [68.1%]) working in physician-owned practices reported being satisfied with their EHR vs 320 respondents (weighted, 63 988 respondents [58.5%]) working in non–physician-owned practices (*P* = .03). In addition, 289 respondents (weighted, 57 702 respondents [36.0%]) working in physician-owned practices had staff support for documentation compared with 146 respondents (weighted, 29 267 respondents [26.7% ]) working in non–physician-owned practices (*P* = .02). Overall, 328 respondents (weighted, 71 827 respondents [44.8%]) working in physician-owned practices perceived that time spent on clinical documentation was appropriate compared with 191 respondents (weighted, 35 447 respondents [32.4%]) working in non–physician-owned practices (*P* = .005). There were no significant differences by practice ownership in the ease of documentation, the distribution of hours per day spent on documentation outside of normal office hours, or the perceptions that time spent on documentation reduced time with patients or that time spent on billing increased documentation time.

**Table 3.** Unadjusted EHR Experiences By Practice Ownership

Experience	Physician-owned		Non–physician-owned		*P* value
	Unweighted, No.	Weighted, No. (%)	Unweighted, No.	Weighted, No. (%)	
Total participants, No.	766	161 226	602	109 587	NA
Satisfied with EHR	529	108 093 (68.1)	320	63 988 (58.5)	.03
Staff support (eg, scribes) for documentation is available	289	57 702 (36.0)	146	29 267 (26.7)	.02
Time spent on documentation is appropriate	328	71 827 (44.8)	191	35 447 (32.4)	.005
Time spent on documentation does not reduce time spent with patients	318	66 761 (41.5)	207	40 323 (36.8)	.28
Documenting in medical record system is very or somewhat easy	518	107 108 (66.8)	331	64 530 (59.2)	.08
Time spent on billing increases documentation time	625	127 867 (83.7)	514	92 160 (86.9)	.36
Mean time spent outside of normal office hours documenting clinical care, h					
None	57	11 185 (6.9)	40	7147 (6.5)	.64
<1	134	23 754 (14.7)	102	20 863 (19.0)	
1 to 2	323	72 076 (44.7)	232	42 893 (39.1)	
>2 to <4	189	39 205 (24.3)	168	28 873 (26.3)	
>4	63	15 005 (9.3)	60	9784 (8.9)	

Abbreviations: EHR, electronic health record; NA, not applicable.

### Analyses Stratified by Specialty

When stratified by specialty, primary care clinicians working in physician-owned practices (267 of 356 physicians; weighted, 48 587 of 66 389 physicians [73.2%]) were significantly more satisfied with their EHR compared with their counterparts practicing in locations not owned by a physician (185 of 342 physicians; weighted, 39 441 of 64 786 physicians [60.9%]; *P* = .04) (Figure, panel A). In addition, surgical clinicians in physician-owned vs non–physician-owned practices were significantly more likely to perceive that time spent on clinical documentation was appropriate (82 of 192 physicians; weighted, 21 073 of 43 110 physicians [48.9%] vs 38 of 105 physicians; weighted, 4428 of 16 226 physicians [27.3%]; *P* = .01) (Figure, panel B) and to have staff support for documentation (106 of 191 physicians; weighted, 22 819 of 43 053 physicians [54.3%] vs 33 of 108 physicians; weighted, 4546 of 16 330 physicians [27.8%]; *P* = .006) (Figure, panel C). Among surgical and medical specialists, EHR satisfaction did not vary significantly by ownership type; among primary care physicians or medical specialists, staff support for documentation and perceptions of the appropriateness of time spent on documentation did not vary significantly by ownership type.

**Figure.**
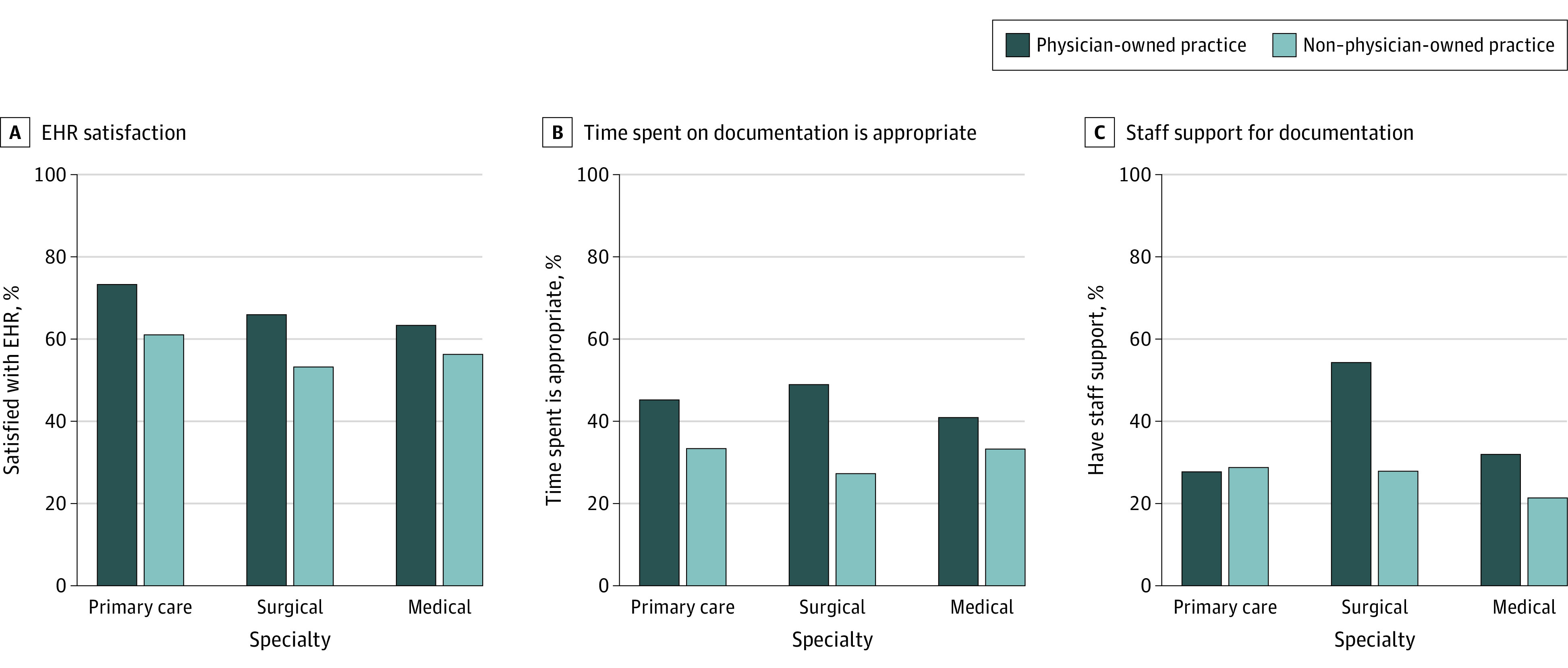
Electronic Health Record (EHR) Satisfaction, Staff Support, and Documentation Perceptions for Physician-Owned vs Non–Physician-Owned Practices, Stratified by Specialty

### Multivariable Analyses

In multivariable analysis adjusted for physician specialty and ACO status, significant differences in EHR satisfaction persisted among physicians working in physician-owned vs non–physician-owned practices (Table 4). Those working in physician-owned practices were more likely to be satisfied with their EHR than their counterparts working in non–physician-owned practices (odds ratio [OR], 1.60; 95% CI, 1.10-2.32; *P* = .01). Practice size and EHR vendor were collinear with ownership categories; thus, they were not included as control variables in our main multivariable analyses. Although there were differences in EHR satisfaction by vendor (eTable 1 in the Supplement), the significant association between ownership type and EHR satisfaction persisted after adjustment for EHR vendor (eTable 2 in the Supplement), both in our main model (OR, 2.16; 95% CI, 1.36-3.43; *P* = .001) and in models adjusting for additional covariates (eg, adjusted for ownership type, EHR vendor, specialty, ACO status, and practice size: OR, 2.29; 95% CI, 1.42-3.68; *P* = .001). Perceptions of time spent on documentation and staff support for documentation partially explained the association between ownership type and EHR satisfaction, with respondents from physician-owned practices more likely to be satisfied than respondents from non–physician-owned practices after adjustment for each of these differences (Table 4).

**Table 4.** Models Estimating Association Between Practice Ownership and EHR Satisfaction

Model	EHR satisfaction in physician-owned vs non–physician-owned practices, OR (95% CI)	*P* value
Practice ownership alone	1.52 (1.05-2.19)	.03
Adjusted for specialty	1.61 (1.11-2.33)	.01
Adjusted for specialty and ACO participation	1.60 (1.10-2.32)	.01
Adjusted for specialty, ACO participation, and time spent on documentation	1.42 (0.96-2.09)	.08
Adjusted for specialty, ACO participation, and staff support for documentation	1.59 (1.09-2.32)	.02

Abbreviations: ACO, accountable care organization; EHR, electronic health record; OR, odds ratio.

## Discussion

In this nationally representative cross-sectional study, office-based physicians working in physician-owned practices were significantly more likely to be satisfied with their EHR system, to perceive that time spent on documentation was appropriate, and to have staff support for documentation than those working in non–physician-owned practices. Differences in EHR satisfaction and perceptions of time spent on documentation also varied by specialty. Primary care physicians in physician-owned practices were significantly more likely to be satisfied with their EHR, whereas surgical specialists in physician-owned practices were more likely to perceive that time spent on documentation was appropriate and to have staff support for documentation. These associations were notable given the known associations between EHR usability and burnout^15^ and the impact of EHR experiences for physician satisfaction.^18^

We found that the differences in EHR satisfaction by ownership type partially explained the perceptions that time spent on documentation was appropriate and that staff support for documentation was available. These findings are consistent with physicians working in physician-owned practices being more likely to benefit directly from any increased revenues associated with documentation and from the financial incentives associated with EHR use. In addition, physicians working in physician-owned practices were likely more able to control the tradeoff between their take-home pay and investment in resources to help with documentation.

However, differences in perceptions of documentation time and staff support for documentation did not fully explain the differences in EHR satisfaction by ownership type. In addition, there were no differences by ownership type in several other satisfaction parameters or in time spent using the EHR. This finding suggests that differences in EHR satisfaction are not fully explained by the presence of concrete support resources for documentation, the absolute time spent on documentation, or other experiences of documentation. Rather, it is possible that the cultural and structural attributes of a practice play a role in creating a culture within physician- or physician group–owned practices that allows greater input regarding EHR-related choices and more control over the workplace and how time is spent, producing higher satisfaction.^19^

These findings are consistent with previous research reporting that greater physician autonomy and control were associated with greater satisfaction.^18^ They are also concordant with recent findings by Edwards et al^19^ revealing that zero-burnout practices were more likely to be physician-owned or solo practices. These practices were also more likely to have an environment characterized by strong leadership, effective teamwork, psychological safety, good communication, and a culture of learning. A recent qualitative report of physician-owned practices described the autonomy, pride in work, and sense of control felt by physicians practicing in high-functioning physician-owned practices.^24^ Ongoing policy and market trends resulting in physician group consolidation^25^ or acquisition of physician groups by private equity firms^26^ or health care systems^27^ may limit the ability to preserve cultures that feature high levels of physician control over their work environment.

There were notable cross-specialty differences in EHR satisfaction and documentation perceptions in our study. Primary care physicians working in physician-owned compared with non–physician-owned practices were significantly more likely to be satisfied with their EHR. Given that primary care clinicians spend the most total and after-hours time using the EHR across all specialties,^11^ this difference has important implications for the experiences of primary care physicians working in physician-owned vs non–physician-owned practices. In our analysis, surgical specialists working in physician-owned practices were significantly more likely to perceive that time spent on documentation was appropriate and to have staff support for documentation than those working in non–physician-owned practices. Given their higher mean earnings, surgical specialists working in physician-owned practices may be able to translate the greater decision-making authority associated with practice ownership into concrete resources, such as staff support. In addition, they may be more likely to feel that the time spent on documentation adequately translates into remuneration and is thus appropriate.

The EHR vendor used may also play a role in time spent, although we found that vendor choice was associated with practice size. Epic, the most widely used EHR, specifically targets the largest practice groups, and the vendor typically does not sell to very small groups. In addition, we found that physicians in smaller practice groups used a wider range of EHR vendors than physicians in large practice groups. Our analyses revealed that differences in EHR satisfaction by ownership type persisted even after adjustment for EHR vendor in the sensitivity analyses.

### Strengths and Limitations

This study has strengths. First, we used data from a large nationally representative database, for which more than half of the data were derived from physician- or physician group–owned practices and more than 70% of data were derived from practices with 10 or fewer physicians. Although these groups comprise most US ambulatory physicians, their interactions with the EHR have not been well described in the literature; the present study thus increases our understanding of how most US ambulatory physicians currently experience the EHR. Second, we were able to segment the population of interest across multiple characteristics, including practice ownership type and size, physician specialty, and EHR vendor used, which provided enhanced insight into the characteristics that may shape EHR experience.

This study also has limitations. First, the ambulatory and office-based focus of the NEHRS restricts our understanding of the impact of and experience with EHRs to the outpatient setting. The NEHRS relies heavily on self-reporting, including self-reporting of practice characteristics. Although this reliance on self-reporting facilitated an understanding of physician perceptions of the EHR, which is the focus of this study, it may have introduced error in our understanding of the capabilities and availability of resources in different practices. Second, given the design and focus of the NEHRS, it is not possible to control for physicians’ general satisfaction or to exclude the possibility that physicians are unhappy with the tasks they are completing in the EHR rather than with the EHR itself. Third, there were response rate limitations to the 2019 NEHRS, and we do not know whether response rates varied by ownership type. However, the NEHRS specifically weights responses at the physician level to provide nationally representative estimates, and the 59.5% of respondents working in physician-owned practices in our study is generally consistent with the American Medical Association report of 54% of physicians working in physician-owned practices in 2018.^28^ Fourth, we report associations so cannot reach causal conclusions based on our data.

## Conclusions

In this cross-sectional study, physicians working in physician-owned practices were significantly more likely to be satisfied with their EHR system, to perceive that time spent on documentation was appropriate, and to have staff support for documentation compared with those working in non–physician-owned practices. The association between practice ownership and EHR satisfaction was only partially explained by perceptions that the time spent on documentation was appropriate and that staff support for documentation was available. These findings suggest that while some differences in satisfaction are associated with physician-owned practices having resources to help with documentation and being able to experience the results of their documentation more concretely, it is likely that other cultural structure, practice structure, and EHR design choices play a substantial role in determining EHR satisfaction. Future studies may seek to further describe the cultural structure, practice structure, workflow, and EHR design characteristics associated with the differences in EHR satisfaction and support identified in the current study and evaluate whether specific approaches result in better quality and value of care. These studies may include more in-depth explorations of practice culture and the resulting sense of agency, which may be associated with the differences in EHR satisfaction identified. Deeper understanding of these factors may be beneficial in enhancing the physician experience of providing care and in using resources to support physicians in their use of the EHR.

## Notes

Supplement. **eTable 1.** Differences in EHR Satisfaction by EHR Vendor and Practice Ownership **eTable 2.** Models Estimating Associations Between EHR Satisfaction, Practice Ownership, and EHR Vendor
