# Supplementary material for: Assessment of Satisfaction With the Electronic Health Record Among Physicians in Physician-Owned vs Non–Physician-Owned Practices
Source: JAMA Netw Open. 2022 Apr 21;5(4):e228301. doi: 10.1001/jamanetworkopen.2022.8301 (PMC9024386; doi:10.1001/jamanetworkopen.2022.8301)
Supplement: Supplement. — eTable 1. Differences in EHR Satisfaction by EHR Vendor and Practice Ownership eTable 2. Models Estimating Associations Between EHR Satisfaction, Practice Ownership, and EHR Vendor [file jamanetwopen-e228301-s001.pdf]

Supplementary Online Content

Rotenstein LS, Apathy N, Landon B, Bates DW. Assessment of satisfaction with the electronic health record among physicians in physician-owned vs non–physician-owned practices. *JAMA Netw Open*. 2022;5(4):e228301. doi:10.1001/jamanetworkopen.2022.8301

eTable 1. Differences in EHR Satisfaction by EHR Vendor and Practice Ownership

eTable 2. Models Estimating Associations Between EHR Satisfaction, Practice Ownership, and EHR Vendor

This supplementary material has been provided by the authors to give readers additional information about their work.

© 2022 Rotenstein LS et al. *JAMA Network Open*.

eTable 1. Differences in EHR Satisfaction by EHR Vendor and Practice Ownership

	**EHR Satisfaction**		
**EHR Vendor**	**Overall N (%)**	**Physician-Owned N (%)**	**Non-Physician Owned N (%)**
Allscripts	54 (50.8)	44 (58.8)	10 (28.5)
Athenahealth	66 (76.9)	43 (80.2)	23 (67.7)
Cerner	43 (38.7)	7 (38.2)	36 (38.7)
eClinicalWorks	84 (72.3)	71 (79.4)	13 (56.4)
Epic	234 (75.0)	60 (77.1)	174 (74.3)
NextGen	40 (63.2)	32 (79.0)	8 (30.3)
Practice Fusion	29 (82.4)	25 (82.5)	4 (81.9)
Other EHR	295 (~~82.4~~58.2)	244 (61.8)	51 (43.6)

© 2022 Rotenstein LS et al. *JAMA Network Open*.

eTable 2. Models Estimating Associations Between EHR Satisfaction, Practice Ownership, and EHR Vendor

	OR (95% CI) for EHR Satisfaction for Physician-Owned versus Non-Physician Owned	p-value
**Ownership Alone**	1.52 (1.04, 2.19)	0.03
**Ownership and EHR Vendor**	2.16 (1.36, 3.43)	0.001
**Ownership, EHR Vendor and Specialty**	2.21 (1.39, 3.52)	0.001
**Ownership, EHR Vendor, Specialty, and ACO Status**	2.20 (1.38, 3.51)	0.001
**Ownership, EHR Vendor, Specialty, ACO Status, and Practice Size**	2.29 (1.42, 3.68)	0.001

© 2022 Rotenstein LS et al. *JAMA Network Open*.
